# Bis(nitrato-κ*O*)tetra­kis­[1-phenyl-3-(1*H*-1,2,4-triazol-1-yl)propan-1-one]copper(II)

**DOI:** 10.1107/S160053681103529X

**Published:** 2011-09-14

**Authors:** Hua Cai, Ying Guo, Jian-Gang Li, Yao Wu

**Affiliations:** a College of Science, Civil Aviation University of China, Tianjin 300300, People’s Republic of China

## Abstract

In the title complex, [Cu(NO_3_)_2_(C_11_H_11_N_3_O)_4_], the Cu^II^ atom is situated on a centre of inversion and is coordinated by two O atoms from two nitrate anions and four N atoms from four monodentate 1-phenyl-3-(1*H*-1,2,4-triazol-1-yl)propan-1-one ligands in a distorted octa­hedral geometry. Weak inter­molecular C—H⋯O and C—H⋯N hydrogen bonds result in a supra­molecular layer parallel to (101). These layers are connected by π–π inter­actions between the benzene rings [centroid–centroid distance = 3.891 (2) Å].

## Related literature

For background to complexes with neutral N-containing ligands, see: Barnett & Champness (2003 ▶); Roesky & Andruh (2003 ▶); Zaworotko (2001 ▶). For a related structure, see: Cai *et al.* (2010 ▶).
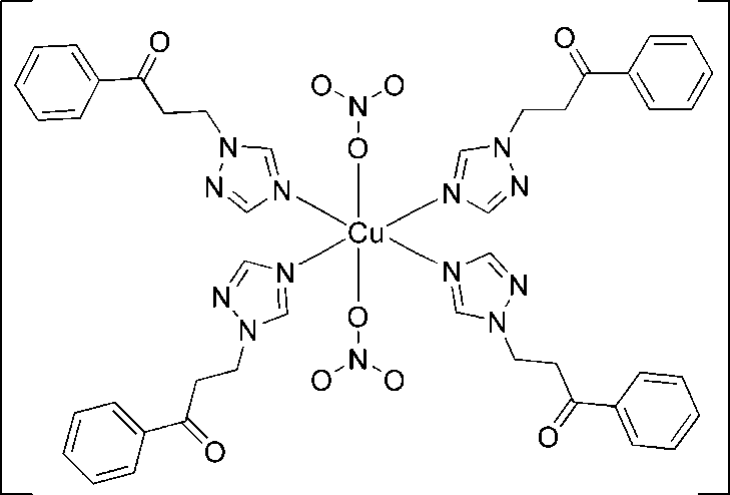


## Experimental

### 

#### Crystal data

[Cu(NO_3_)_2_(C_11_H_11_N_3_O)_4_]
*M* *_r_* = 992.47
Triclinic, 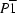

*a* = 7.7742 (14) Å
*b* = 12.472 (2) Å
*c* = 12.498 (2) Å
α = 102.232 (3)°
β = 100.737 (3)°
γ = 104.394 (3)°
*V* = 1110.0 (3) Å^3^
*Z* = 1
Mo *K*α radiation
μ = 0.57 mm^−1^
*T* = 296 K
0.24 × 0.20 × 0.14 mm

#### Data collection

Bruker APEXII CCD diffractometer
Absorption correction: multi-scan (*SADABS*; Sheldrick, 1996 ▶) *T*_min_ = 0.876, *T*_max_ = 0.925
5737 measured reflections
3905 independent reflections
3295 reflections with *I* > 2σ(*I*)
*R*_int_ = 0.016

#### Refinement

*R*[*F* ^2^ > 2σ(*F* ^2^)] = 0.040
*wR*(*F* ^2^) = 0.105
*S* = 1.03
3905 reflections
313 parameters
H-atom parameters constrained
Δρ_max_ = 0.55 e Å^−3^
Δρ_min_ = −0.34 e Å^−3^

### 

Data collection: *APEX2* (Bruker, 2007 ▶); cell refinement: *SAINT* (Bruker, 2007 ▶); data reduction: *SAINT*; program(s) used to solve structure: *SHELXS97* (Sheldrick, 2008 ▶); program(s) used to refine structure: *SHELXL97* (Sheldrick, 2008 ▶); molecular graphics: *SHELXTL* (Sheldrick, 2008 ▶) and *DIAMOND* (Brandenburg & Berndt, 1999 ▶); software used to prepare material for publication: *SHELXTL*.

## Supplementary Material

Crystal structure: contains datablock(s) global, I. DOI: 10.1107/S160053681103529X/hy2463sup1.cif

Structure factors: contains datablock(s) I. DOI: 10.1107/S160053681103529X/hy2463Isup2.hkl

Additional supplementary materials:  crystallographic information; 3D view; checkCIF report

## Figures and Tables

**Table 1:** Hydrogen-bond geometry (Å, °)

*D*—H⋯*A*	*D*—H	H⋯*A*	*D*⋯*A*	*D*—H⋯*A*
C14—H14*A*⋯N2^i^	0.97	2.50	3.368 (4)	148
C19—H19⋯O5^ii^	0.93	2.56	3.291 (4)	136

Symmetry codes: (i) 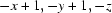; (ii) 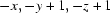.

**Fig. 1.**
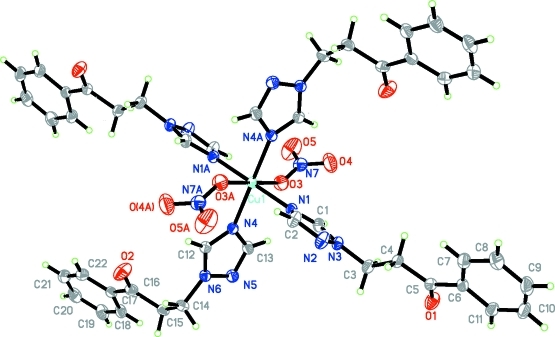
The molecular structure of the title compound. Displacement ellipsoids are drawn at the 30% probability level. [Symmetry code: (A) -x, 1-y, -z.]

**Fig. 2.**
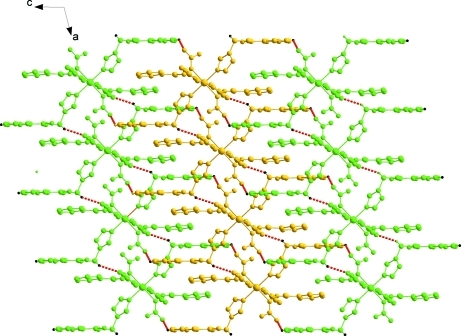
The two-dimensional layer structure of the title compound, showing C—H···O and C—H···N hydrogen bonds as red dashed lines.

## supplementary crystallographic information

### Comment

Neutral organic ligands containing rigid or flexible spacers, such as 4,4'-bipyridine, 1,2-bis(4-pyridyl)ethane, 1,2-bis(4-pyridyl)propane and many others, have been used to generate a rich variety of metal-organic architectures with different metal ions by various reaction procedures (Barnett & Champness, 2003; Roesky & Andruh, 2003; Zaworotko, 2001). Recently, we have initiated a research program of synthesizing supermolecules based on pseudohalides and a flexible ligand, which consists of a propanone unit substituted with a triazole and a phenyl group (Cai *et al.*, 2010). To further explore this series, we synthesized the title compound, a new Cu(II) complex based on the ligand 1-phenyl-3-(1*H*-1,2,4-triazol-1-yl)propan-1-one (*L*).

In the neutral mononuclear title complex (Fig. 1), the Cu^II^ atom is six-coordinated by four monodentate *L* ligands in the equatorial plane and two O atoms from two NO_3_^-^ anions in the axial positions, displaying a CuN_4_O_2_ octahedral geometry. The triazol and phenyl rings in the ligands are not coplanar. The dihedral angels formed by the least-squares planes of the phenyl and triazole rings are 43.8 (2) and 65.9 (2)°. Weak intermolecular C—H···O and C—H···N  hydrogen bonds (Table 1) extend the monomeric units into a two-dimensional supramolecular layer parallel to (1 0 1), as shown in Fig. 2.

### Experimental

Cu(NO_3_)_2_.3H_2_O (24.2 mg, 0.1 mmol) and *L* (22.3 mg, 0.1 mmol) were mixed in a CH_3_CN/H_2_O (20 ml, v/v 1:1) solution with vigorous stirring for *ca* 30 min. The resulting solution was filtered and left to stand at room temperature. Colourless block crystals of the title compound suitable for X-ray analysis were obtained in 65% yield by slow evaporation of the solvent over a period of 1 week. Analysis, calculated for C_46_H_44_MnN_14_O_4_S_2_: C 53.25, H 4.47, N 19.76%; found: C 53.45, H 4.43, N 19.62%.

### Refinement

Although all H atoms were visible in difference Fourier maps, they were finally placed in geometrically calculated positions and refined as riding atoms, with C—H = 0.93 (aromatic) and 0.97 (methylene) Å and with *U*_iso_(H) = 1.2*U*_eq_(C).

### Crystal data

**Table:**

[Cu(NO_3_)_2_(C_11_H_11_N_3_O)_4_]	*Z* = 1
*M**_r_* = 992.47	*F*(000) = 515
Triclinic, *P*1	*D*_x_ = 1.485 Mg m^−^^3^
Hall symbol: -P 1	Mo *K*α radiation, λ = 0.71073 Å
*a* = 7.7742 (14) Å	Cell parameters from 2167 reflections
*b* = 12.472 (2) Å	θ = 2.8–25.5°
*c* = 12.498 (2) Å	µ = 0.57 mm^−^^1^
α = 102.232 (3)°	*T* = 296 K
β = 100.737 (3)°	Block, blue
γ = 104.394 (3)°	0.24 × 0.20 × 0.14 mm
*V* = 1110.0 (3) Å^3^	

### Data collection

**Table:**

Bruker APEXII CCD diffractometer	3905 independent reflections
Radiation source: fine-focus sealed tube	3295 reflections with *I* > 2σ(*I*)
graphite	*R*_int_ = 0.016
φ and ω scans	θ_max_ = 25.0°, θ_min_ = 1.8°
Absorption correction: multi-scan (*SADABS*; Sheldrick, 1996)	*h* = −9→9
*T*_min_ = 0.876, *T*_max_ = 0.925	*k* = −14→10
5737 measured reflections	*l* = −13→14

### Refinement

**Table:**

Refinement on *F*^2^	Primary atom site location: structure-invariant direct methods
Least-squares matrix: full	Secondary atom site location: difference Fourier map
*R*[*F*^2^ > 2σ(*F*^2^)] = 0.040	Hydrogen site location: inferred from neighbouring sites
*wR*(*F*^2^) = 0.105	H-atom parameters constrained
*S* = 1.03	*w* = 1/[σ^2^(*F*_o_^2^) + (0.0485*P*)^2^ + 0.6085*P*] where *P* = (*F*_o_^2^ + 2*F*_c_^2^)/3
3905 reflections	(Δ/σ)_max_ < 0.001
313 parameters	Δρ_max_ = 0.55 e Å^−^^3^
0 restraints	Δρ_min_ = −0.34 e Å^−^^3^

### Fractional atomic coordinates and isotropic or equivalent isotropic displacement parameters (Å^2^)

**Table:**

	*x*	*y*	*z*	*U*_iso_*/*U*_eq_	
Cu1	0.0000	0.5000	0.0000	0.03192 (15)	
O1	0.1209 (3)	−0.14388 (17)	−0.15924 (18)	0.0600 (6)	
O2	0.6023 (4)	0.8407 (2)	0.42668 (19)	0.0725 (7)	
O3	−0.2044 (3)	0.38551 (18)	0.0827 (2)	0.0617 (6)	
O4	−0.4197 (4)	0.2377 (2)	−0.0202 (2)	0.0874 (8)	
O5	−0.4769 (4)	0.3628 (3)	0.1018 (3)	0.1086 (11)	
N1	0.0657 (3)	0.36119 (17)	−0.06904 (17)	0.0338 (5)	
N2	0.2167 (4)	0.26287 (19)	−0.1668 (2)	0.0493 (6)	
N3	0.1188 (3)	0.19722 (17)	−0.11162 (17)	0.0351 (5)	
N4	0.1884 (3)	0.51953 (16)	0.14694 (16)	0.0335 (5)	
N5	0.3189 (3)	0.47333 (18)	0.29969 (18)	0.0414 (5)	
N6	0.4316 (3)	0.56936 (17)	0.28594 (17)	0.0330 (5)	
N7	−0.3682 (3)	0.3303 (2)	0.0570 (2)	0.0495 (6)	
C1	0.0307 (4)	0.2570 (2)	−0.0540 (2)	0.0374 (6)	
H1	−0.0444	0.2297	−0.0097	0.045*	
C2	0.1795 (4)	0.3605 (2)	−0.1381 (2)	0.0468 (7)	
H2	0.2276	0.4237	−0.1633	0.056*	
C3	0.1336 (4)	0.0823 (2)	−0.1151 (2)	0.0426 (6)	
H3A	0.0389	0.0418	−0.0848	0.051*	
H3B	0.2518	0.0884	−0.0681	0.051*	
C4	0.1139 (4)	0.0150 (2)	−0.2340 (2)	0.0397 (6)	
H4A	0.2116	0.0543	−0.2630	0.048*	
H4B	−0.0020	0.0122	−0.2815	0.048*	
C5	0.1208 (3)	−0.1061 (2)	−0.2409 (2)	0.0382 (6)	
C6	0.1233 (3)	−0.1786 (2)	−0.3521 (2)	0.0376 (6)	
C7	0.0983 (4)	−0.1428 (2)	−0.4503 (2)	0.0486 (7)	
H7	0.0843	−0.0704	−0.4472	0.058*	
C8	0.0941 (4)	−0.2141 (3)	−0.5531 (3)	0.0581 (8)	
H8	0.0746	−0.1901	−0.6187	0.070*	
C9	0.1190 (4)	−0.3198 (3)	−0.5576 (3)	0.0610 (9)	
H9	0.1160	−0.3677	−0.6264	0.073*	
C10	0.1481 (4)	−0.3550 (2)	−0.4609 (3)	0.0581 (9)	
H10	0.1668	−0.4264	−0.4644	0.070*	
C11	0.1500 (4)	−0.2856 (2)	−0.3582 (3)	0.0461 (7)	
H11	0.1692	−0.3105	−0.2932	0.055*	
C12	0.3529 (3)	0.5953 (2)	0.1955 (2)	0.0332 (6)	
H12	0.4050	0.6574	0.1698	0.040*	
C13	0.1750 (4)	0.4467 (2)	0.2141 (2)	0.0412 (6)	
H13	0.0727	0.3835	0.2006	0.049*	
C14	0.6175 (3)	0.6206 (2)	0.3603 (2)	0.0400 (6)	
H14A	0.6857	0.6797	0.3316	0.048*	
H14B	0.6794	0.5618	0.3585	0.048*	
C15	0.6195 (4)	0.6732 (2)	0.4821 (2)	0.0383 (6)	
H15A	0.5158	0.6264	0.5009	0.046*	
H15B	0.7306	0.6721	0.5317	0.046*	
C16	0.6107 (4)	0.7953 (2)	0.5032 (2)	0.0409 (6)	
C17	0.6149 (3)	0.8587 (2)	0.6201 (2)	0.0382 (6)	
C18	0.6220 (4)	0.8080 (2)	0.7086 (2)	0.0445 (7)	
H18	0.6255	0.7325	0.6964	0.053*	
C19	0.6238 (4)	0.8694 (3)	0.8155 (3)	0.0580 (8)	
H19	0.6280	0.8348	0.8745	0.070*	
C20	0.6193 (4)	0.9811 (3)	0.8344 (3)	0.0600 (9)	
H20	0.6210	1.0220	0.9062	0.072*	
C21	0.6123 (4)	1.0324 (3)	0.7475 (3)	0.0601 (9)	
H21	0.6091	1.1081	0.7605	0.072*	
C22	0.6099 (4)	0.9717 (2)	0.6406 (3)	0.0504 (7)	
H22	0.6049	1.0069	0.5819	0.060*	

### Atomic displacement parameters (Å^2^)

**Table:**

	*U*^11^	*U*^22^	*U*^33^	*U*^12^	*U*^13^	*U*^23^
Cu1	0.0358 (3)	0.0315 (2)	0.0255 (2)	0.01221 (18)	0.00439 (18)	0.00191 (17)
O1	0.0937 (17)	0.0467 (12)	0.0502 (13)	0.0292 (12)	0.0234 (12)	0.0209 (10)
O2	0.126 (2)	0.0701 (15)	0.0463 (13)	0.0520 (15)	0.0302 (14)	0.0325 (12)
O3	0.0424 (12)	0.0555 (13)	0.0895 (17)	0.0057 (10)	0.0197 (11)	0.0325 (12)
O4	0.095 (2)	0.0634 (16)	0.0724 (18)	−0.0020 (14)	−0.0014 (15)	0.0032 (14)
O5	0.102 (2)	0.094 (2)	0.170 (3)	0.0450 (18)	0.096 (2)	0.048 (2)
N1	0.0401 (12)	0.0319 (11)	0.0282 (11)	0.0125 (9)	0.0091 (9)	0.0031 (9)
N2	0.0711 (17)	0.0410 (13)	0.0495 (15)	0.0233 (12)	0.0348 (13)	0.0159 (11)
N3	0.0445 (12)	0.0296 (11)	0.0316 (11)	0.0116 (9)	0.0123 (10)	0.0063 (9)
N4	0.0382 (12)	0.0321 (11)	0.0281 (11)	0.0103 (9)	0.0050 (9)	0.0070 (9)
N5	0.0489 (13)	0.0389 (12)	0.0352 (13)	0.0097 (10)	0.0065 (11)	0.0155 (10)
N6	0.0361 (11)	0.0338 (11)	0.0277 (11)	0.0090 (9)	0.0071 (9)	0.0079 (9)
N7	0.0480 (15)	0.0492 (15)	0.0577 (16)	0.0122 (12)	0.0174 (13)	0.0272 (13)
C1	0.0429 (15)	0.0340 (14)	0.0332 (14)	0.0084 (11)	0.0137 (12)	0.0050 (11)
C2	0.069 (2)	0.0383 (15)	0.0432 (16)	0.0201 (14)	0.0271 (15)	0.0153 (12)
C3	0.0554 (17)	0.0328 (14)	0.0414 (16)	0.0169 (12)	0.0116 (13)	0.0100 (12)
C4	0.0471 (15)	0.0319 (13)	0.0381 (15)	0.0149 (12)	0.0068 (12)	0.0054 (11)
C5	0.0360 (14)	0.0334 (13)	0.0428 (16)	0.0097 (11)	0.0065 (12)	0.0096 (12)
C6	0.0321 (13)	0.0309 (13)	0.0446 (16)	0.0066 (10)	0.0067 (12)	0.0055 (11)
C7	0.0565 (18)	0.0384 (15)	0.0459 (17)	0.0142 (13)	0.0092 (14)	0.0044 (13)
C8	0.061 (2)	0.0571 (19)	0.0442 (18)	0.0099 (16)	0.0091 (15)	0.0020 (15)
C9	0.0533 (19)	0.0520 (19)	0.061 (2)	0.0097 (15)	0.0167 (16)	−0.0149 (16)
C10	0.0476 (17)	0.0343 (15)	0.086 (3)	0.0137 (13)	0.0185 (17)	−0.0007 (16)
C11	0.0393 (15)	0.0345 (14)	0.064 (2)	0.0112 (12)	0.0145 (14)	0.0115 (13)
C12	0.0392 (14)	0.0333 (13)	0.0278 (13)	0.0104 (11)	0.0094 (11)	0.0097 (10)
C13	0.0470 (16)	0.0364 (14)	0.0347 (15)	0.0060 (12)	0.0066 (12)	0.0090 (11)
C14	0.0334 (14)	0.0492 (16)	0.0356 (15)	0.0142 (12)	0.0054 (11)	0.0087 (12)
C15	0.0376 (14)	0.0430 (15)	0.0302 (14)	0.0095 (12)	0.0012 (11)	0.0110 (11)
C16	0.0413 (15)	0.0482 (16)	0.0348 (15)	0.0152 (12)	0.0050 (12)	0.0166 (12)
C17	0.0333 (13)	0.0407 (14)	0.0349 (15)	0.0078 (11)	0.0016 (11)	0.0089 (11)
C18	0.0502 (16)	0.0390 (15)	0.0350 (15)	0.0042 (12)	0.0039 (12)	0.0076 (12)
C19	0.066 (2)	0.0575 (19)	0.0357 (17)	0.0012 (16)	0.0055 (15)	0.0091 (14)
C20	0.057 (2)	0.066 (2)	0.0393 (18)	0.0092 (16)	0.0051 (15)	−0.0056 (15)
C21	0.060 (2)	0.0468 (17)	0.061 (2)	0.0198 (15)	−0.0002 (16)	−0.0022 (15)
C22	0.0531 (18)	0.0471 (17)	0.0475 (18)	0.0177 (14)	−0.0001 (14)	0.0138 (14)

### Geometric parameters (Å, °)

**Table:**

Cu1—N1	1.9931 (19)	C6—C11	1.389 (4)
Cu1—N4	2.0515 (19)	C7—C8	1.388 (4)
Cu1—O3	2.396 (2)	C7—H7	0.9300
O1—C5	1.211 (3)	C8—C9	1.370 (4)
O2—C16	1.210 (3)	C8—H8	0.9300
O3—N7	1.232 (3)	C9—C10	1.370 (5)
O4—N7	1.257 (3)	C9—H9	0.9300
O5—N7	1.201 (3)	C10—C11	1.384 (4)
N1—C1	1.321 (3)	C10—H10	0.9300
N1—C2	1.346 (3)	C11—H11	0.9300
N2—C2	1.313 (3)	C12—H12	0.9300
N2—N3	1.360 (3)	C13—H13	0.9300
N3—C1	1.325 (3)	C14—C15	1.520 (4)
N3—C3	1.459 (3)	C14—H14A	0.9700
N4—C12	1.328 (3)	C14—H14B	0.9700
N4—C13	1.360 (3)	C15—C16	1.512 (4)
N5—C13	1.313 (3)	C15—H15A	0.9700
N5—N6	1.361 (3)	C15—H15B	0.9700
N6—C12	1.324 (3)	C16—C17	1.496 (4)
N6—C14	1.467 (3)	C17—C18	1.385 (4)
C1—H1	0.9300	C17—C22	1.391 (4)
C2—H2	0.9300	C18—C19	1.387 (4)
C3—C4	1.503 (4)	C18—H18	0.9300
C3—H3A	0.9700	C19—C20	1.373 (5)
C3—H3B	0.9700	C19—H19	0.9300
C4—C5	1.510 (3)	C20—C21	1.371 (5)
C4—H4A	0.9700	C20—H20	0.9300
C4—H4B	0.9700	C21—C22	1.384 (4)
C5—C6	1.495 (4)	C21—H21	0.9300
C6—C7	1.389 (4)	C22—H22	0.9300
			
N1—Cu1—N1^i^	180.00 (11)	C11—C6—C5	119.3 (3)
N1—Cu1—N4^i^	91.68 (8)	C8—C7—C6	120.7 (3)
N1^i^—Cu1—N4^i^	88.32 (8)	C8—C7—H7	119.7
N1—Cu1—N4	88.32 (8)	C6—C7—H7	119.7
N1^i^—Cu1—N4	91.68 (8)	C9—C8—C7	119.8 (3)
N4^i^—Cu1—N4	180.00 (11)	C9—C8—H8	120.1
N1—Cu1—O3^i^	90.37 (8)	C7—C8—H8	120.1
N1^i^—Cu1—O3^i^	89.63 (8)	C10—C9—C8	120.1 (3)
N4^i^—Cu1—O3^i^	82.97 (8)	C10—C9—H9	119.9
N4—Cu1—O3^i^	97.03 (8)	C8—C9—H9	119.9
N1—Cu1—O3	89.63 (8)	C9—C10—C11	120.6 (3)
N1^i^—Cu1—O3	90.37 (8)	C9—C10—H10	119.7
N4^i^—Cu1—O3	97.03 (8)	C11—C10—H10	119.7
N4—Cu1—O3	82.97 (8)	C10—C11—C6	120.1 (3)
O3^i^—Cu1—O3	180.0	C10—C11—H11	120.0
N7—O3—Cu1	136.91 (19)	C6—C11—H11	120.0
C1—N1—C2	103.6 (2)	N6—C12—N4	109.9 (2)
C1—N1—Cu1	132.14 (18)	N6—C12—H12	125.1
C2—N1—Cu1	123.99 (17)	N4—C12—H12	125.1
C2—N2—N3	102.5 (2)	N5—C13—N4	114.2 (2)
C1—N3—N2	110.0 (2)	N5—C13—H13	122.9
C1—N3—C3	129.9 (2)	N4—C13—H13	122.9
N2—N3—C3	119.9 (2)	N6—C14—C15	113.0 (2)
C12—N4—C13	103.1 (2)	N6—C14—H14A	109.0
C12—N4—Cu1	131.17 (17)	C15—C14—H14A	109.0
C13—N4—Cu1	125.54 (17)	N6—C14—H14B	109.0
C13—N5—N6	102.6 (2)	C15—C14—H14B	109.0
C12—N6—N5	110.2 (2)	H14A—C14—H14B	107.8
C12—N6—C14	130.0 (2)	C16—C15—C14	113.0 (2)
N5—N6—C14	119.5 (2)	C16—C15—H15A	109.0
O5—N7—O3	122.4 (3)	C14—C15—H15A	109.0
O5—N7—O4	120.4 (3)	C16—C15—H15B	109.0
O3—N7—O4	117.3 (3)	C14—C15—H15B	109.0
N1—C1—N3	109.7 (2)	H15A—C15—H15B	107.8
N1—C1—H1	125.2	O2—C16—C17	121.1 (3)
N3—C1—H1	125.2	O2—C16—C15	120.1 (3)
N2—C2—N1	114.3 (2)	C17—C16—C15	118.8 (2)
N2—C2—H2	122.8	C18—C17—C22	118.7 (3)
N1—C2—H2	122.8	C18—C17—C16	122.1 (2)
N3—C3—C4	111.1 (2)	C22—C17—C16	119.1 (2)
N3—C3—H3A	109.4	C17—C18—C19	120.3 (3)
C4—C3—H3A	109.4	C17—C18—H18	119.8
N3—C3—H3B	109.4	C19—C18—H18	119.8
C4—C3—H3B	109.4	C20—C19—C18	120.2 (3)
H3A—C3—H3B	108.0	C20—C19—H19	119.9
C3—C4—C5	112.3 (2)	C18—C19—H19	119.9
C3—C4—H4A	109.1	C21—C20—C19	120.1 (3)
C5—C4—H4A	109.1	C21—C20—H20	119.9
C3—C4—H4B	109.1	C19—C20—H20	119.9
C5—C4—H4B	109.1	C20—C21—C22	120.0 (3)
H4A—C4—H4B	107.9	C20—C21—H21	120.0
O1—C5—C6	121.2 (2)	C22—C21—H21	120.0
O1—C5—C4	120.9 (2)	C21—C22—C17	120.6 (3)
C6—C5—C4	118.0 (2)	C21—C22—H22	119.7
C7—C6—C11	118.6 (3)	C17—C22—H22	119.7
C7—C6—C5	122.1 (2)		
			
N1—Cu1—O3—N7	−92.4 (3)	C3—C4—C5—C6	−173.5 (2)
N1^i^—Cu1—O3—N7	87.6 (3)	O1—C5—C6—C7	171.1 (3)
N4^i^—Cu1—O3—N7	−0.8 (3)	C4—C5—C6—C7	−7.5 (4)
N4—Cu1—O3—N7	179.2 (3)	O1—C5—C6—C11	−8.4 (4)
N4^i^—Cu1—N1—C1	−107.5 (2)	C4—C5—C6—C11	172.9 (2)
N4—Cu1—N1—C1	72.5 (2)	C11—C6—C7—C8	2.1 (4)
O3^i^—Cu1—N1—C1	169.5 (2)	C5—C6—C7—C8	−177.5 (3)
O3—Cu1—N1—C1	−10.5 (2)	C6—C7—C8—C9	−1.5 (5)
N4^i^—Cu1—N1—C2	79.8 (2)	C7—C8—C9—C10	−0.1 (5)
N4—Cu1—N1—C2	−100.2 (2)	C8—C9—C10—C11	1.0 (5)
O3^i^—Cu1—N1—C2	−3.2 (2)	C9—C10—C11—C6	−0.4 (4)
O3—Cu1—N1—C2	176.8 (2)	C7—C6—C11—C10	−1.2 (4)
C2—N2—N3—C1	−0.2 (3)	C5—C6—C11—C10	178.4 (2)
C2—N2—N3—C3	−175.6 (2)	N5—N6—C12—N4	0.2 (3)
N1—Cu1—N4—C12	107.1 (2)	C14—N6—C12—N4	173.7 (2)
N1^i^—Cu1—N4—C12	−72.9 (2)	C13—N4—C12—N6	−0.2 (3)
O3^i^—Cu1—N4—C12	16.9 (2)	Cu1—N4—C12—N6	−175.26 (16)
O3—Cu1—N4—C12	−163.1 (2)	N6—N5—C13—N4	−0.1 (3)
N1—Cu1—N4—C13	−66.9 (2)	C12—N4—C13—N5	0.2 (3)
N1^i^—Cu1—N4—C13	113.1 (2)	Cu1—N4—C13—N5	175.61 (17)
O3^i^—Cu1—N4—C13	−157.1 (2)	C12—N6—C14—C15	118.9 (3)
O3—Cu1—N4—C13	22.9 (2)	N5—N6—C14—C15	−68.1 (3)
C13—N5—N6—C12	0.0 (3)	N6—C14—C15—C16	−85.4 (3)
C13—N5—N6—C14	−174.4 (2)	C14—C15—C16—O2	0.3 (4)
Cu1—O3—N7—O5	−107.0 (4)	C14—C15—C16—C17	−178.9 (2)
Cu1—O3—N7—O4	72.1 (4)	O2—C16—C17—C18	179.1 (3)
C2—N1—C1—N3	−0.2 (3)	C15—C16—C17—C18	−1.7 (4)
Cu1—N1—C1—N3	−173.99 (16)	O2—C16—C17—C22	−0.3 (4)
N2—N3—C1—N1	0.3 (3)	C15—C16—C17—C22	179.0 (2)
C3—N3—C1—N1	175.1 (2)	C22—C17—C18—C19	0.1 (4)
N3—N2—C2—N1	0.0 (3)	C16—C17—C18—C19	−179.3 (3)
C1—N1—C2—N2	0.1 (3)	C17—C18—C19—C20	−0.2 (5)
Cu1—N1—C2—N2	174.55 (19)	C18—C19—C20—C21	0.2 (5)
C1—N3—C3—C4	137.6 (3)	C19—C20—C21—C22	−0.1 (5)
N2—N3—C3—C4	−48.0 (3)	C20—C21—C22—C17	−0.1 (5)
N3—C3—C4—C5	−177.6 (2)	C18—C17—C22—C21	0.1 (4)
C3—C4—C5—O1	7.8 (4)	C16—C17—C22—C21	179.5 (3)

Symmetry codes: (i) −*x*, −*y*+1, −*z*.

### Hydrogen-bond geometry (Å, °)

**Table:**

*D*—H···*A*	*D*—H	H···*A*	*D*···*A*	*D*—H···*A*
C14—H14A···N2^ii^	0.97	2.50	3.368 (4)	148.
C19—H19···O5^iii^	0.93	2.56	3.291 (4)	136.

Symmetry codes: (ii) −*x*+1, −*y*+1, −*z*; (iii) −*x*, −*y*+1, −*z*+1.
